# Supplementary material for: Liquid foam improves potency and safety of gene therapy vectors
Source: Nat Commun. 2024 May 28;15:4523. doi: 10.1038/s41467-024-48753-9 (PMC11133309; doi:10.1038/s41467-024-48753-9)
Supplement: Supplementary file 1 — Supplementary Information [file 41467_2024_48753_MOESM1_ESM.pdf]

Supplementary Information

Liquid foam improves potency and safety of gene therapy vectors

Fitzgerald, et al.

•Size Distribution (Nanosight): 92.3 ± 1.6 nm
•Zeta Potential: 4.5 ± 2.2 mV
•MRNA Encapsulation: 98-99%

Batch #1

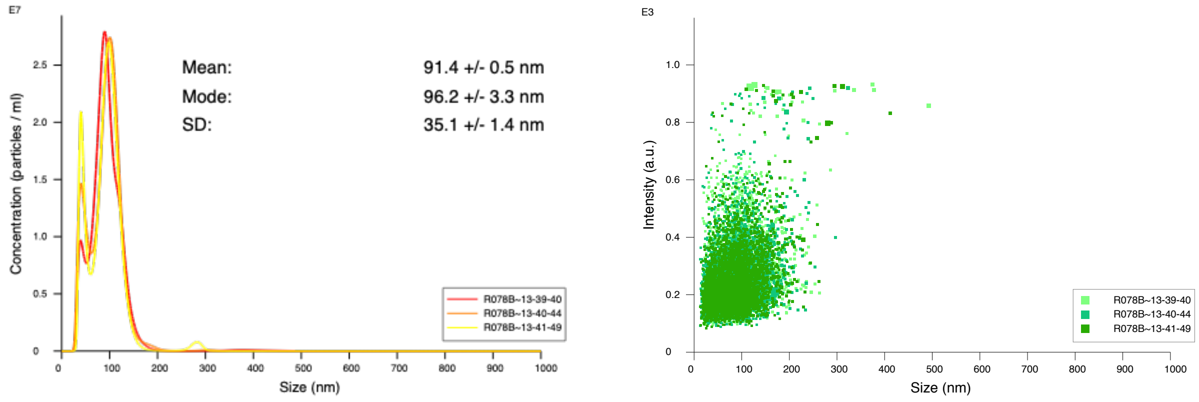

Batch #2

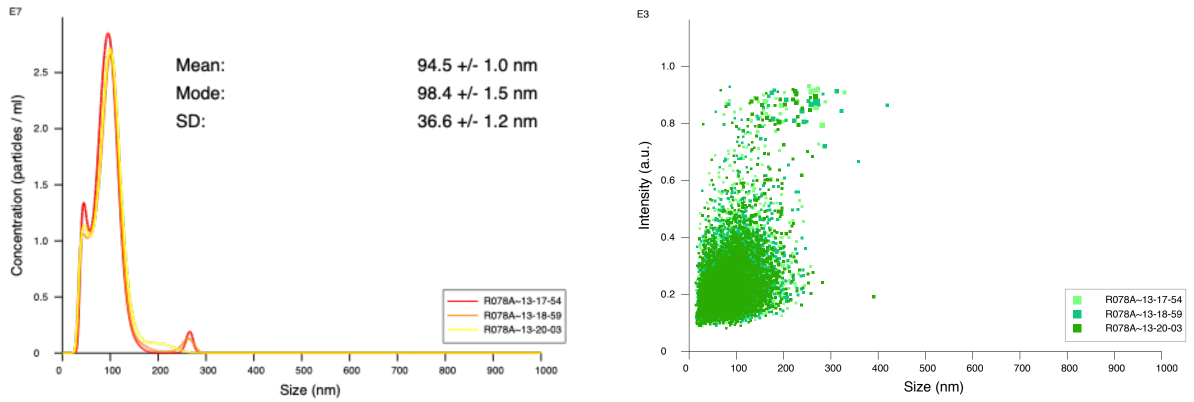

Batch #3

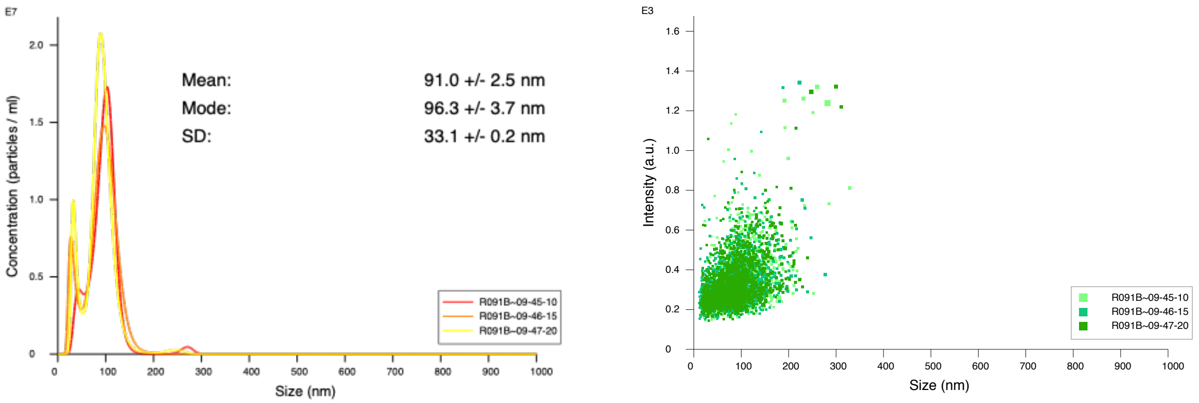

Supplementary Figure 1: Physicochemical characterization of Lipid nanoparticles (LNPs). Size distribution was measured via Nanoparticle Tracking Analysis using a NanoSight NS300 (Malvern) and zeta potential was determined using dynamic light scattering detected with a ZetaPALS instrument (Brookhaven). The particles were diluted 1:100 (v/v) in PBS for size measurements, and 1:33 (v/v) in water for zeta potential quantitation. Size distribution was determined for three independently prepared batches of LNPs. For each batch, three consecutive videos (60 s each) were captured under constant flow conditions. MRNA encapsulation efficiency was determined using a Qubit RNA High Sensitivity Assay Kit (Invitrogen). LNPs were incubated with the Qubit RNA reagent in the presence and absence of 1% Triton X-100. Fluorescence intensities for total mRNA after release from LNPs by Triton X-100 were compared to fluorescence intensities for unencapsulated mRNA measured in the absence of Triton X-100.

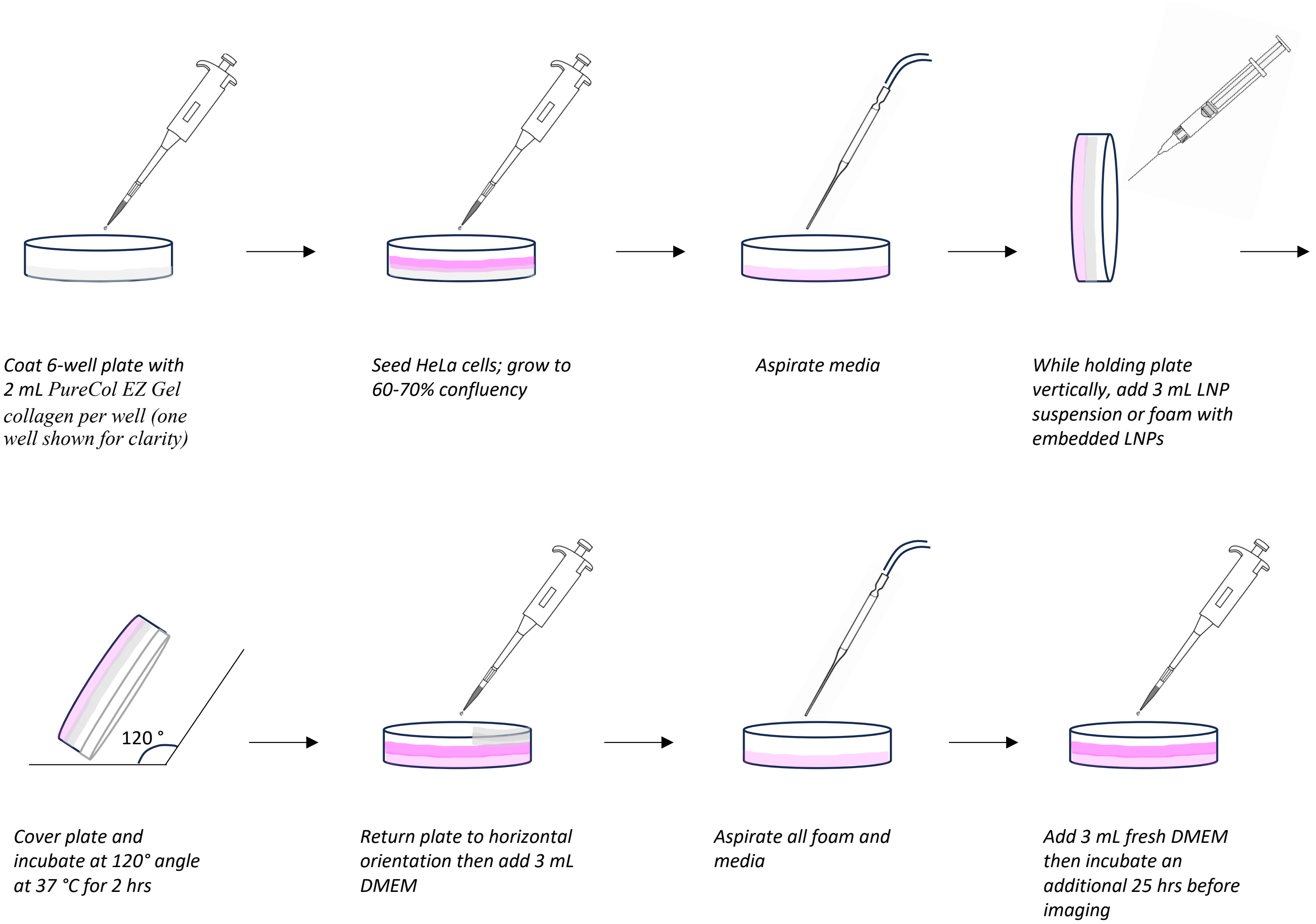

Supplementary Figure 2: Diagrammatic illustration of "angled transfections" (performed in Fig. 2a, b). Created with BioRender.com.

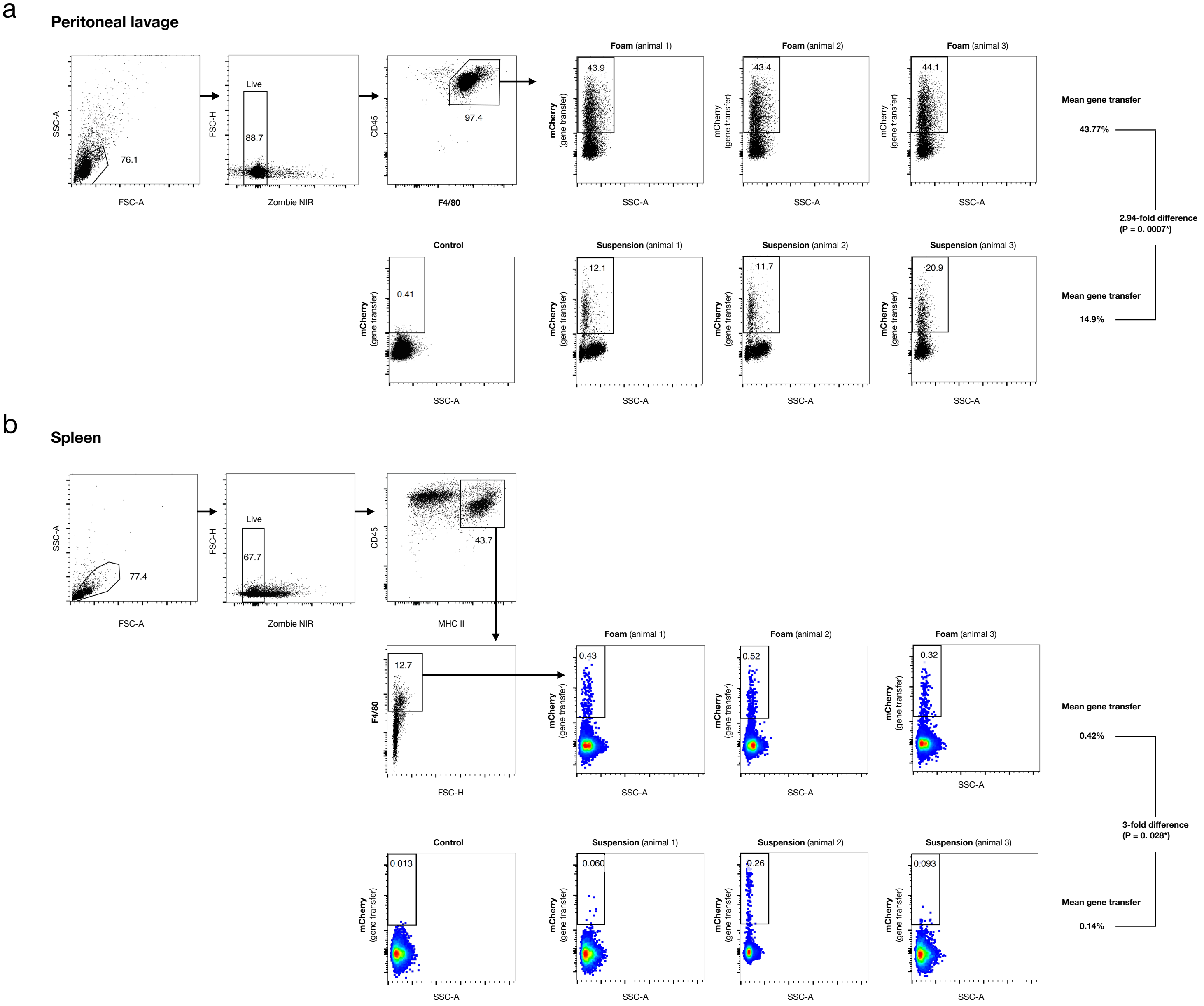

Supplementary Figure 3: Flow cytometric quantification of rates of transfection into peritoneal and splenic macrophages in vivo using foam versus suspension. Immunocompetent C57BL/6 albino mice were injected intraperitoneally with a single dose of LNPs suspended in PBS or incorporated into methylcellulose foam. Controls were injected with PBS. To track gene expression by flow cytometry, LNPs were loaded with mRNA encoding mCherry (10 µg/mouse). Twenty-four hours after injection, peritoneal exudate cells and splenocytes were harvested. Flow cytometric quantitation of in vivo transfection rates in macrophages are summarized in (**a**) and (**b**), respectively. Gating strategies for flow cytometric analysis are shown on top of each panel. N = 3 biologically independent samples. $P < 0.05$ was considered significant (*).

Supplementary Table 1: List of primary antibodies and other staining reagents used for flow cytometry

Specificity	Clone	Isotype	Dye	Supplier	Catalog #
Mouse CD45	30-F11	IgG2b kappa	BUV395	BD Biosciences	564279
Mouse MCH Class II	M5/114.15.2	IgG2b kappa	Alexa Fluor 700	Life Technologies	56-5321-82
Mouse F4/80	T45-2342	IgG2a kappa	BUV496	BD Biosciences	750644
LIVE/DEAD Fixable Near-IR	-	-	NIR	Life Technologies	L34976
