# Supplementary material for: Liquid foam improves potency and safety of gene therapy vectors
Source: Nat Commun. 2024 May 28;15:4523. doi: 10.1038/s41467-024-48753-9 (PMC11133309; doi:10.1038/s41467-024-48753-9)
Supplement: Supplementary file 3 — Reporting Summary [file 41467_2024_48753_MOESM3_ESM.pdf]

nature portfolio

Corresponding author(s): Stephan MT

Last updated by author(s): 2024/05/10

Reporting Summary

Nature Portfolio wishes to improve the reproducibility of the work that we publish. This form provides structure for consistency and transparency in reporting. For further information on Nature Portfolio policies, see our Editorial Policies and the Editorial Policy Checklist.

Statistics

For all statistical analyses, confirm that the following items are present in the figure legend, table legend, main text, or Methods section.

n/a	Confirmed	
☐	☒	The exact sample size (*n*) for each experimental group/condition, given as a discrete number and unit of measurement
☐	☒	A statement on whether measurements were taken from distinct samples or whether the same sample was measured repeatedly
☐	☒	The statistical test(s) used AND whether they are one- or two-sided<br>*Only common tests should be described solely by name; describe more complex techniques in the Methods section.*
☐	☒	A description of all covariates tested
☒	☐	A description of any assumptions or corrections, such as tests of normality and adjustment for multiple comparisons
☐	☒	A full description of the statistical parameters including central tendency (e.g. means) or other basic estimates (e.g. regression coefficient) AND variation (e.g. standard deviation) or associated estimates of uncertainty (e.g. confidence intervals)
☐	☒	For null hypothesis testing, the test statistic (e.g. F, t, r) with confidence intervals, effect sizes, degrees of freedom and P value noted<br>*Give P values as exact values whenever suitable.*
☒	☐	For Bayesian analysis, information on the choice of priors and Markov chain Monte Carlo settings
☒	☐	For hierarchical and complex designs, identification of the appropriate level for tests and full reporting of outcomes
☒	☐	Estimates of effect sizes (e.g. Cohen's *d*, Pearson's *r*), indicating how they were calculated

Our web collection on statistics for biologists contains articles on many of the points above.

Software and code

Policy information about availability of computer code

Data collection	Bioluminescence was acquired using Living Image (R) version 4.7.3 software (Caliper Life Sciences).<br>Dynamic Foam Analyzer DFA100FSM data was acquired using ADVANCE software.<br>Confocal microscopy images were acquired with an Andor Zyla 4.2 Plus sCMOS camera controlled with Fusion software (version 2.4.0.13).<br>TissueFAXS images were collected with a TissueFAXS PLUS digital pathology system/slide scanner, using TissueFAXS Imaging Software (version 7.1).
Data analysis	Bioluminescence images were analyzed using Living Image (R) version 4.7.3 software (Caliper Life Sciences).<br>Dynamic Foam Analyzer DFA100FSM data was analyzed using ADVANCE software.<br>Confocal microscopy images were analyzed using ImageJ (Version 1.53t).<br>TissueFAXS data was analyzed using TissueFAXS Imaging Software (version 7.1) and ImageJ (Version 1.53t).

For manuscripts utilizing custom algorithms or software that are central to the research but not yet described in published literature, software must be made available to editors and reviewers. We strongly encourage code deposition in a community repository (e.g. GitHub). See the Nature Portfolio guidelines for submitting code & software for further information.

Data

Policy information about availability of data

All manuscripts must include a data availability statement. This statement should provide the following information, where applicable:

- Accession codes, unique identifiers, or web links for publicly available datasets
- A description of any restrictions on data availability
- For clinical datasets or third party data, please ensure that the statement adheres to our policy

All data supporting the findings of this study are available within the article and its supplementary information files. The data generated in this study have been deposited in the Figshare database: Stephan M. T. Liquid foam improves potency and safety of gene therapy vectors. figshare. https://figshare.com/s/3328220d46a16c5a56ed (2024). Source data are provided with this paper.

Research involving human participants, their data, or biological material

Policy information about studies with human participants or human data. See also policy information about sex, gender (identity/presentation), and sexual orientation and race, ethnicity and racism.

Reporting on sex and gender	N/A
Reporting on race, ethnicity, or other socially relevant groupings	N/A
Population characteristics	N/A
Recruitment	N/A
Ethics oversight	N/A

Note that full information on the approval of the study protocol must also be provided in the manuscript.

Field-specific reporting

Please select the one below that is the best fit for your research. If you are not sure, read the appropriate sections before making your selection.

☒ Life sciences ☐ Behavioural & social sciences ☐ Ecological, evolutionary & environmental sciences

For a reference copy of the document with all sections, see nature.com/documents/nr-reporting-summary-flat.pdf

Life sciences study design

All studies must disclose on these points even when the disclosure is negative.

Sample size	We carried out our in vivo studies in 5 or 10 mice per treatment group. In vitro assays were conducted in triplicates and repeated at least twice.
Data exclusions	All data were included in the analysis. No data were excluded from the analysis
Replication	All experiments were replicated at least two times by two independent scientists working as a team on this project. Bioluminescent imaging data was acquired and analyzed by two scientists working on this project. Foam analysis and characterization was conducted at Kruess Scientific (www.kruss-scientific.com) by an independent scientist and replicated three times. All replication attempts were successful.
Randomization	Mice were randomized into the control, foam or PBS suspension treatment groups immediately before gene therapy.
Blinding	Investigators were not blinded, since the key therapeutic readout was bioluminescent tumor imaging, which is an unbiased readout. All toxicity and safety studies (Figure 6) were conducted by a board-certified staff pathologist in a double-blinded fashion.

Reporting for specific materials, systems and methods

We require information from authors about some types of materials, experimental systems and methods used in many studies. Here, indicate whether each material, system or method listed is relevant to your study. If you are not sure if a list item applies to your research, read the appropriate section before selecting a response.

Materials & experimental systems

n/a	Involved in the study
☐	☒ Antibodies
☐	☒ Eukaryotic cell lines
☒	☐ Palaeontology and archaeology
☐	☒ Animals and other organisms
☒	☐ Clinical data
☒	☐ Dual use research of concern
☒	☐ Plants

Methods

n/a	Involved in the study
☒	☐ ChIP-seq
☒	☐ Flow cytometry
☒	☐ MRI-based neuroimaging

Antibodies

Antibodies used

Specificity Clone Isotype Dye Supplier Catalog #
Mouse CD45 30-F11 IgG2b kappa BUV395 BD Biosciences 564279
Mouse CD45* 30-F11 IgG2b kappa PE-Texas Red ThermoFisher MCD4517
Mouse MCH Class II M5/114.15.2 IgG2b kappa Alexa Fluor 700 Life Technologies 56-5321-82
Mouse F4/80 T45-2342 IgG2a kappa BUV496 BD Biosciences 750644
LIVE/DEAD Fixable Near-IR - - NIR Life Technologies L34976

Validation

Describe the validation of each primary antibody for the species and application, noting any validation statements on the manufacturer's website, relevant citations, antibody profiles in online databases, or data provided in the manuscript.

Eukaryotic cell lines

Policy information about cell lines and Sex and Gender in Research

Cell line source(s)

HeLa cells for in vitro transfection assays were obtained from ATCC (Cat# CCL-2)

Authentication

Raji lymphoma was directly ordered from ATCC. No additional cell authentication was performed.

Mycoplasma contamination

Cells tested negative for mycoplasma using a DNA-based PCR test (DDC Medical).

Commonly misidentified lines
(See ICLAC register)

No commonly misidentified cells lines were used in this study.

Animals and other research organisms

Policy information about studies involving animals; ARRIVE guidelines recommended for reporting animal research, and Sex and Gender in Research

Laboratory animals

Four to six-week-old female albino B6 (C57BL/6J-Tyr<c-2J>) mice (Strain #:000058) used in all in vivo experiments were obtained from Jackson Laboratory.

Wild animals

The study did not involve wild animals.

Reporting on sex

We used female mice for intraperitoneal foam/suspension injections as male mice of these strain showed aggressive behavior between cage mates. Given the large number of animals/treatment arm single-housing animals was not an option.

Field-collected samples

The study did not involve samples collected from the field.

Ethics oversight

The care and use of mice in this study was approved by the Institutional Animal Care & Use Committee (IACUC) at the Fred Hutchinson Cancer Research Center, and was in compliance with all relevant ethical regulations for animal testing and research (Assurance #A3226-01, IACUC Protocol Number 50782).

Note that full information on the approval of the study protocol must also be provided in the manuscript.

Plants

Seed stocks	N/A
Novel plant genotypes	N/A
Authentication	N/A
